# Effect of white-coat hypertension on arterial stiffness: A meta-analysis

**DOI:** 10.1097/MD.0000000000012888

**Published:** 2018-10-19

**Authors:** Peng Cai, Yan Peng, Yan Wang, Xukai Wang

**Affiliations:** a Department of Cardiology, Institute of Field Surgery, Daping Hospital, Third Military Medical University, Chongqing; b Key Laboratory of Basic Pharmacology of Ministry of Education Joint International Research Laboratory of Ministry Education, Zunyi Medical University, Zunyi, China.

**Keywords:** arterial stiffness, cardio-cerebrovascular disease, isolated clinic hypertension, pulse wave velocity, white-coat hypertension

## Abstract

**Background:**

White-coat hypertension (WCH) is a debatable risk factor of cardio-cerebrovascular diseases and the current study results on the association between WCH and arterial stiffness are inconsistent. The aim was to investigate the effect of WCH on arterial stiffness using meta-analysis.

**Methods:**

Based on prespecified search strategies and inclusion criteria, Medline, Embase, Web Of Science, Cochrane Library, and BioSciences Information Service Preview databases were reviewed. A total of 20 studies involving 1538 WCH patients and 3582 normotensives (NT) were included. Literatures were screened for data extraction and quality assessment. Overall analysis and subgroup analysis were conducted in RevMan version 5.3 and Stata version 14.0 software.

**Results:**

Overall analysis showed that carotid-femoral pulse wave velocity (cf-PWV) was significantly higher in WCH group than in the NT group (*P* < .00001, 95% CI: 0.79–3.26). Subgroup analysis showed that in adults, cf-PWV was significantly higher in the WCH patients than in the NT subjects (*P*<.001, 95% CI: 0.46–0.87), while in juveniles, cf-PWV was comparable between the WCH group and the NT group (*P* = .25, 95% CI: −0.39 to 0.61).

**Conclusion:**

This meta-analysis showed that WCH may increase arterial stiffness in adult population.

## 1 Introduction

White-coat hypertension (WCH), also termed isolated clinic hypertension, is seen in the patients who show hypertension during the clinic visits.^[1]^ Currently, the diagnostic criteria of hypertension has been updated and the diagnostic criteria of WCH vary by guidelines.^[2,3]^ The widely used traditional criteria defines WCH as:

Clinic systolic blood pressure ≥140 mm Hg and/or diastolic pressure ≥90 mm Hg, and mean ambulatory blood pressure <135/85 mm Hg daytime or home blood pressure <135/85 mm Hg. WCH was once considered a benign phenomenon, but several studies have established its relationship with multiple metabolic disorders such as impaired glucose tolerance, insulin resistance, and metabolic syndrome.^[4,5]^ Ongoing studies have been directed to clarify the role of WCH in cardio-cerebrovascular impairments.^[6]^

Arterial stiffness examination is a noninvasive tool to evaluate cardio-cerebrovascular risks. Many clinical studies and basic researches have revealed arterial stiffness as a risk factor of cardio-cerebrovascular diseases. With the popularization of arterial stiffness examination, some indicators such as pulse wave velocity (PWV), ambulatory arterial stiffness index (AASI), and augmentation index have been developed. Of note, both American Heart Association scientific statement and European expert consensus have recommended PWV as the golden standard for arterial stiffness with consideration to its high accuracy and applicability.^[7]^ To identify the target organs of WCH in cardio-cerebrovascular impairments, several clinical studies have attempted to investigate the relationship between WCH and arterial stiffness. However, their results vary due to confounding factors such as small sample size, racial difference, inconsistent methods, and discrepant inclusion criteria.^[8]^ In light of the inconsistencies of relationship between WCH and arterial stiffness, this systematic review and meta-analysis were conducted to evaluate the relationship between WCH and arterial stiffness.

## 2 Methods

### 2.1 Search strategies

Medline, Embase, Web Of Science, Cochrane Library, and BioSciences Information Service (BIOSIS) Preview databases were searched using the combination of text words and keywords of the following terms: “clinic hypertension, “office hypertension,” “white-coat,” “PWV,” “pulse wave velocity,” “arterial stiffness,” “aortic stiffness,” and “vascular stiffness.” Publication date was limited to December 23, 2017.

#### 2.1.1 Inclusion criteria

1. Arterial stiffness measured by cf-PWV;
2. Case-control studies including WCH group and NT group;
3. WCH was defined as an office BP ≥140/90 mm Hg with day ABPM <135/85 mm Hg.

**Figure 1:**
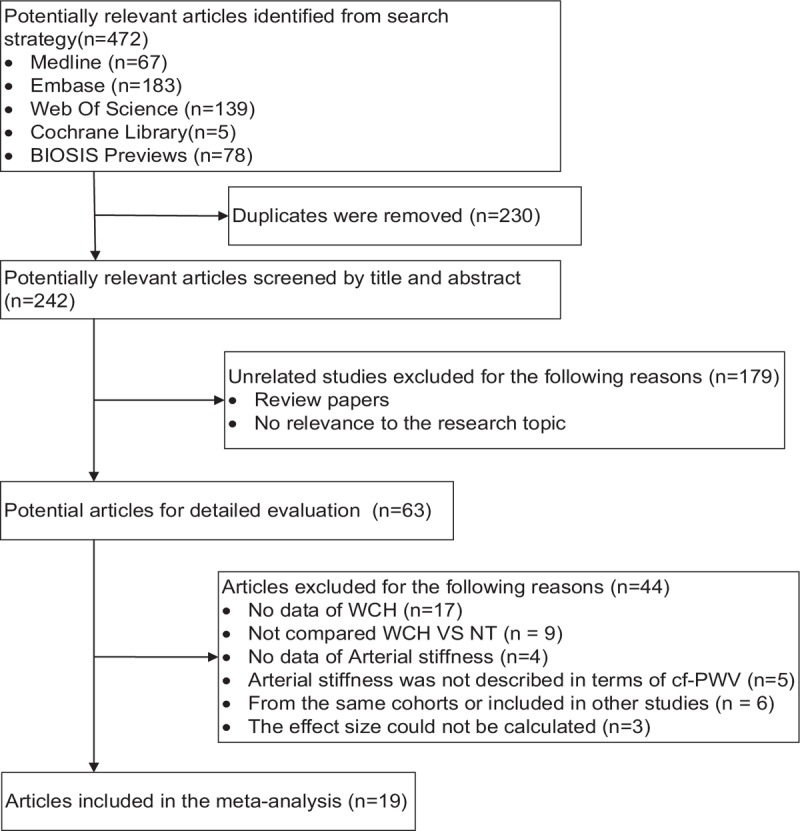
Flow of articles through review.

Literatures of the same study population, poor research quality, and incomplete data reporting were excluded. If a paper included several independent case-control groups, they were screened and the eligible ones were included in the meta-analysis. Figure 1 shows the flowchart of study design.

### 2.2 Data extraction and quality assessment

Two investigators (PC and YP) independently searched literature, screened studies, and extracted data on the basis of searched strategies, and inclusion criteria. The quality of studies was assessed by population selection, comparability between cases and controls, and exposure measurement in accordance with the Newcastle-Ottawa Quality Assessment Scale (NOS). The NOS contains 8 items with a maximum score of 9 points. All studies were classified as low quality (0–3 points), medium quality (4–6 points), or high quality (7–9 points) based on NOS.^[9]^

### 2.3 Statistical analysis

The cf-PWV was compared between WCH group and NT group. All statistical analyses were conducted in RevMan software version 5.3 (The Cochrane Collaboration, Copenhagen, Denmark) and Stata version 14.0 (Stata Corp LP, College Station, TX). All the data were calculated for their 95% confidence intervals (95% CI). Statistical difference was defined as a 2-sided *P* value equal to or smaller than .05.

All the data were transformed into mean ± standard deviation format by either RevMan version 5.3 software or manual calculation. Publication bias analysis, sensitivity analysis, heterogeneity analysis, data synthesis, Z test, meta-regression analysis, and subgroup analysis were performed. Publication bias was analyzed with Begg and Egger tests and visually examined by funnel plot. Sensitivity analysis was performed with Cohen test and graphical methods. Twelve was used to quantitatively assess heterogeneity. When significant heterogeneity was indicated by I2>50%, the random-effects model was used to calculate effect size; otherwise, fixed-effects model was used, followed by Z test. Subgroup analysis was performed for age, blood pressure, instrument for inspecting PWV, history of diabetes mellitus and/or cardiovascular diseases, and quality score. For patients without history of diabetes mellitus and cardiovascular diseases, we further conducted subgroup analysis by antihypertensive treatments, meta-regression analysis was conducted in Stata version 14.0 to identify the sources of heterogeneity. All analyses were based on previous published studies, thus no ethical approval and patient consent are required.

## 3 Results

### 3.1 Studies retrieved and characteristics

A total of 472 articles were retrieved from Medline, Embase, Web Of Science, Cochrane Library, and BIOSIS Preview databases. After duplicate removal, the articles were screened by title, abstract and then full-text, thus 19 articles were finally included. The eligible articles included 5120 subjects (WCH group: 1538, NT group: 3582) from 20 studies and 12 countries. Baseline characteristics varied by study. Two studies included juveniles, while the remaining studies included adults. Only 1 study specifically included antihypertensive drug users, 10 studies specifically included nonantihypertensive drug users, and the remaining studies included mixed users. Regarding comorbidities, 9 studies excluded patients with diabetes mellitus or cardiovascular diseases. NOS score was medium and high in 3 and 17 studies, respectively. Table 1 shows the baseline characteristics.^[10–28]^

**Table 1:**
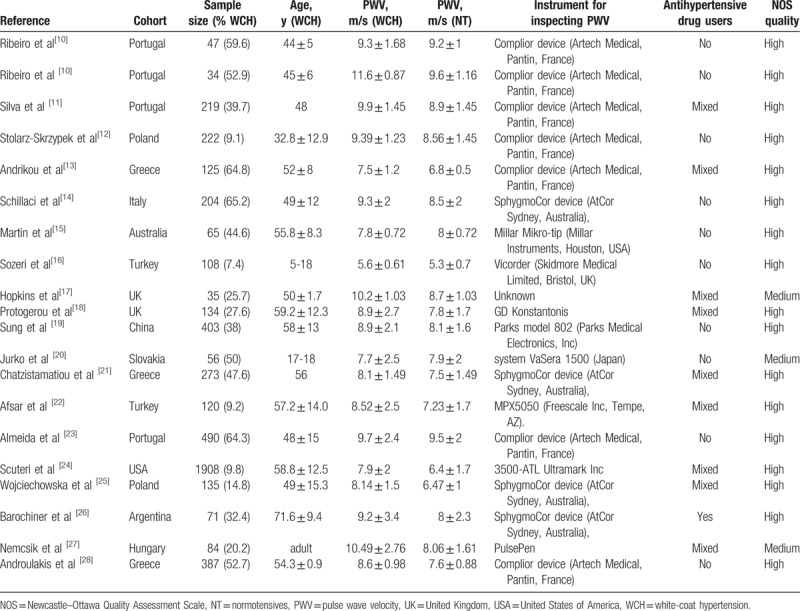
Study characteristics.

### 3.2 Relationship between WCH and PWV

#### 3.2.1 Overall analysis

**Figure 2:**
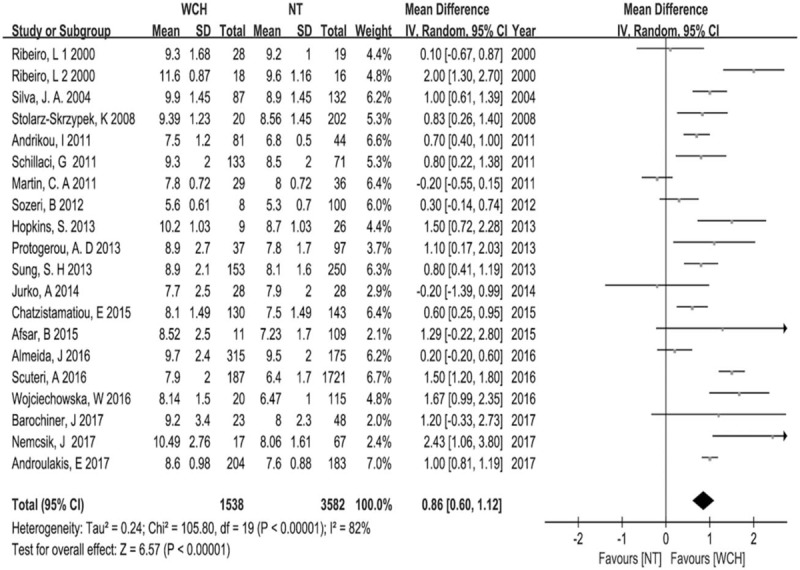
Forest plot of the comparison: white-coat hypertension versus normotension.

**Figure 3:**
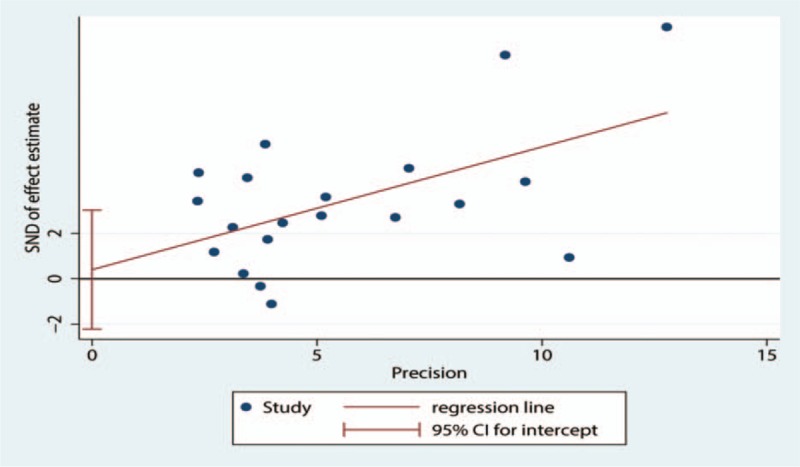
Publication bias. SND = standard normal deviation.

**Figure 4:**
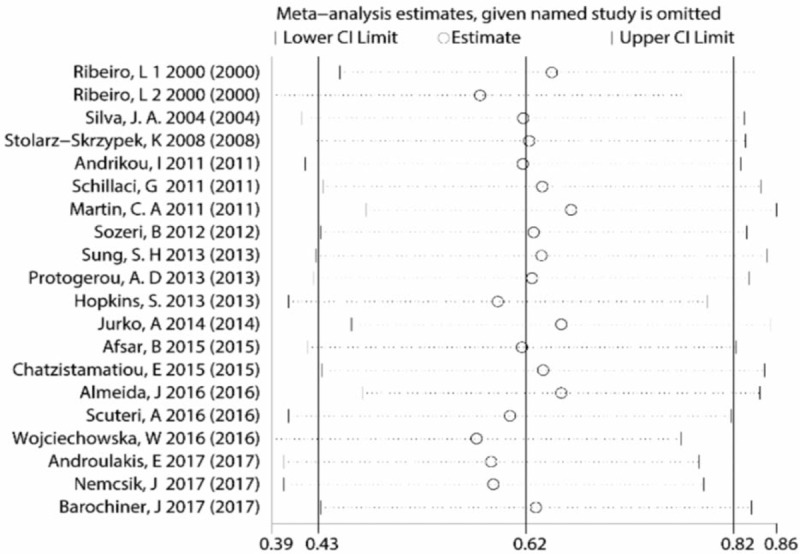
Sensitivity analysis.

Meta-analysis of 20 eligible studies showed cf-PWV was significantly higher in WCH group than in NT group (Z = 6.57, *P* < .00001, 95% CI: 0.79–3.26; Fig. 2), but the heterogeneity was noticeable (I2 = 82%). Egger test and Begg test revealed neither publication bias nor small-study effects (Egger test, *P* = .751; Begg test, *P* = .626), and Fig. 3 visually reflected the publication bias. For random-effects model, sensitivity analysis revealed no significant changes of effect size (Fig. 4). Meta-regression analysis indicated the heterogeneity was partly attributed to comorbidities including diabetes mellitus and cardiovascular diseases (*P* < .05, R-squared = 21.24%).

#### 3.2.2 Subgroup analysis

**Figure 5:**
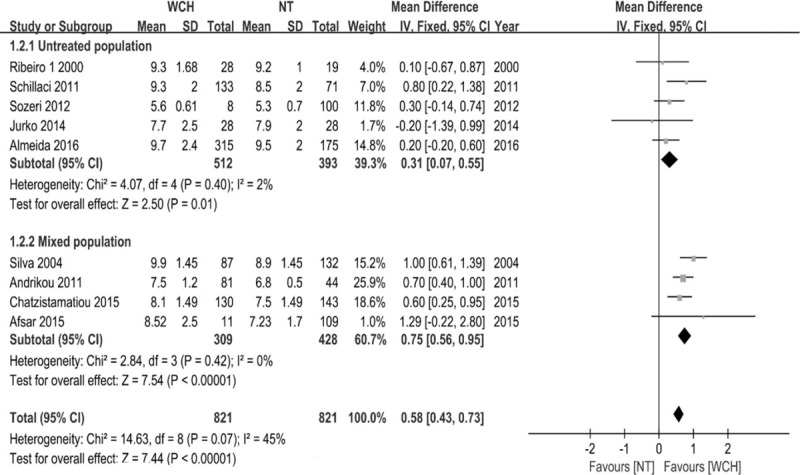
Forrest plot for nondiabetic and noncardiovascular disease population: white-coat hypertension group versus normotension group. Subgroups: 1.2.1 untreated population; 1.2.2 mixed population of treated patients and untreated patients.

The studies were stratified by the history of antihypertensive drug use, age, instrument for inspecting PWV and study quality, and Table 2 shows all subgroup analysis results. For adults, PWV was significantly higher in WCH group than in NT group (*P*<.001, 95% CI: 0.46–0.87), but PWV was not different between WCH group and NT group in juveniles (*P* = .253, 95% CI: −0.39 to 0.61). In the subgroup analysis of 9 studies excluding patients with diabetes mellitus or cardiovascular diseases, heterogeneity was significantly reduced (I2 = 45%; Fig. 5), and PWV differed between WCH group and NT group (*P* < .00001, 95% CI: 0.43–0.73; Fig. 5). When these 9 studies were further divided by history of antihypertensive drug use, untreated group and mixed group showed significantly reduced heterogeneity (I2 = 2%; I2 = 0%; Fig. 5), and PWV differed between WCH group and NT group (*P* = .01, 95% CI: 0.07–0.55; *P* < .00001, 95% CI: 0.56–0.95; Fig. 5).

**Table 2:**
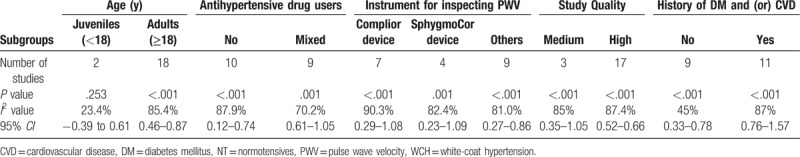
Subgroup analysis (WCH versus NT).

## 4 Discussion

Meta-analysis evaluated the relationship between WCH and arterial stiffness. It was found that adult WCH patients had significantly higher cf-PWV than normal population, indicating higher risks of cardio-cerebrovascular diseases in these patients. However, juveniles did not show the phenomenon, probably attributable to short duration of WCH and a low degree of arterial stiffness. Moreover, only 2 studies containing 164 juveniles were analysed, which might not have fully represented the real situations of juveniles. More studies are needed to reveal the relationship between WCH and arterial stiffness in the juvenile population.

During literature screening, some studies were identified which used AASI and augmentation index to quantify arterial stiffness.^[29,30]^ These accessory examinations have been accepted by clinical practitioners. In particular, AASI calculated from ambulatory blood pressure monitoring is easy to use. Nevertheless, PWV, as the golden standard of arterial stiffness, has a markedly higher diagnostic accuracy than other indexes. Meta-analysis included clinical studies which had employed PWV as an examination method to best show the relationship between WCH and arterial stiffness. Recently, Upala et al^[31]^ published another meta-analysis about the relationship between WCH and arterial stiffness, but they reported no significant association between WCH and arterial hypertension on the basis of 4 eligible observational studies containing persistent hypertension group, WCH group and normal control group. In our opinion, due to their inclusion methods, they might have excluded many case-control studies which only contained WCH group and normal control group thus the study did not sufficiently reveal the relationship between WCH and arterial stiffness. Based on prespecified search strategies and inclusion criteria, Medline, Embase, Web Of Science, Cochrane Library, and BIOSIS Preview databases were reviewed. A total of 20 studies involving 1538 WCH patients and 3582 normotensives were included in our study, which would better reflect the effect of WCH on arterial stiffness.

A limitation of this meta-analysis is that we had no individual patient data, only the literature data can be combined and analyzed. A further limitation was that the heterogeneity for overall analysis was noticeable (I2 = 82%), so the meta-regression analysis and subgroup analysis were performed. Subgroup analysis is the highlight of meta-analysis, especially that of the patients without diabetes mellitus or cardiovascular diseases. Maine–Syracuse case-control study has demonstrated the significant relationship between type-2 diabetes mellitus (especially uncontrolled type-2 diabetes mellitus) and arterial stiffness. Previous studies have proven the close relationship between cardiovascular diseases (e.g., coronary artery disease) and arterial stiffness.^[32,33]^ Therefore, subgroup analysis for the patients without diabetes mellitus or cardiovascular diseases was conducted. The results showed significantly reduced heterogeneity in the eligible studies, which was further reduced by the secondary subgroup analysis stratified by history of antihypertensive drug use. In this way, subgroup analyses identified the relationship between WCH and arterial stiffness. By stepwise subgroup analyses, the eligible criteria was gradually narrowed to reduce the heterogeneity and to enhance the reliability of study results. Meta-regression analysis also identified diabetes mellitus and cardiovascular diseases as important sources of overall heterogeneity.

This study showed that WCH may cause arterial stiffness in adult population. This kind of mechanisms may help uncover the multiple target organ damages in the future. WCH is common in clinical practice, but its pathophysiological mechanisms and target organ damages remain unclear. As a result, many clinicians are confused about its diagnosis and treatments. Based on these study findings, more attention is to be given to the role of WCH in cardio-cerebrovascular target organ damages, and reasonable diagnostic and therapeutic standards of WCH should be further explored.

## Author contributions

**Formal analysis:** Yan Wang.

**Funding acquisition:** Xukai Wang.

**Software:** Peng Cai, Yan Peng.

**Writing – review & editing:** Peng Cai, Xukai Wang.
